# Case report: A rare case of neutropenia caused by pembrolizumab in squamous lung cancer and literature review

**DOI:** 10.3389/fonc.2022.973421

**Published:** 2022-11-25

**Authors:** Qiaoyun Tan, Lichao Liu, Yu Huang, Xiaorong Dong, Lingjuan Chen

**Affiliations:** Cancer Center, Union Hospital, Tongji Medical College, Huazhong University of Science and Technology, Wuhan, China

**Keywords:** neutropenia, pembrolizumab, lung cancer, adverse effect, infection

## Abstract

**Background:**

Immune checkpoint inhibitors, including anti-PD-1 therapies, have prolonged overall survival in patients with a variety of cancers, and immunotherapy is sometimes associated with immune-related adverse events (irAEs); however, hematological toxicity, especially neutropenia, is rare.

**Case presentation:**

A 78-year-old man with squamous lung cancer, with brain metastasis, was treated with pembrolizumab and albumin-bound paclitaxel as first-line treatment for one cycle and changed to pembrolizumab plus anlotinib at the second cycle. After two therapy cycles, grade 4 neutropenia developed, which mainly contributed to irAEs. The patient was started on granulocyte colony-stimulating factor (G-CSF) but did not improve; he was then treated with corticosteroids, and neutrophil counts gradually returned to normal levels. However, the patient eventually died because of neurological problems.

**Conclusion:**

Grade 4 neutropenia associated with ICI, although rare, is often severe and presents with infectious complications; it needs to be diagnosed early, and clinicians should ensure prompt and proper management to such patients.

## Background

Immune checkpoint inhibitors (ICIs) including programmed death-1 (PD-1)/programmed cell death-ligand 1 (PD-L1) and cytotoxic t-lymphocyte-associated antigen 4 (CTLA4) antibodies have revolutionized cancer therapy in various cancer types (1). However, it is associated with immune-related adverse events (irAEs), which can sometimes cause serious consequences (2, 3). Hematological irAE is rare following ICI treatment, with a frequency of 3.6% for all grades and 0.7% for grades III–IV (4). The detailed category is as follows: immune thrombocytopenia (ITP), aplastic anemia (AA)/pancytopenia, neutropenia, autoimmune hemolytic anemia (AIHA), and hemophagocytic syndrome (HPS). Immune-related neutropenia accounts for 17% of all hematological irAEs and is one of the rare but severe irAEs for complicated infections following neutropenia. Clinicians need to have an early diagnosis to ensure management of neutropenia and secondary infections. A previous report showed one case of developed *Klebsiella pneumoniae* caused by ICI-related neutropenia (5); here, we report a case of neutropenia induced by pembrolizumab, which was granulocyte-stimulating factor injection (G-CSF)-refractory, followed by bacterial and fungal infection.

## Case presentation

**Figure 1:**
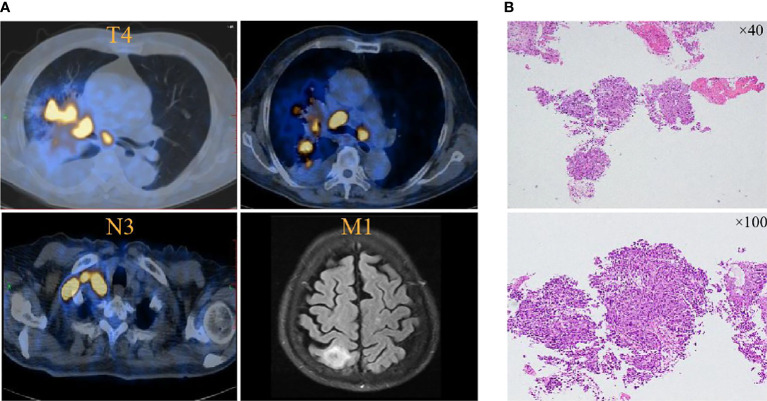
PET and brain MRI images at initial diagnosis. **(A)** Lung cancer is present in the hilar angle of the right lung before pembrolizumab treatment in November 2021. **(B)** Tissue biopsy showed squamous lung cancer.

A 78-year-old man visited the authors’ hospital complaining of chest tightness, cough, sputum, right back pain, and lower limb edema for 2 months; he was later diagnosed with squamous lung cancer of stage IVB (cT4N3M1) with brain metastasis (**Figure 1**). He had hypertension for 30 years, diabetes for more than 20 years, coronary heart disease for 2 years, and colon cancer treated by surgery 6 years ago; hypertension and diabetes were well-controlled by medication and no repeated infectious complications have occurred. He was an ex-smoker with 30 packs per year. The tumor cell proportion score (TPS) for PD-L1 staining was 40%.

**Figure 2:**
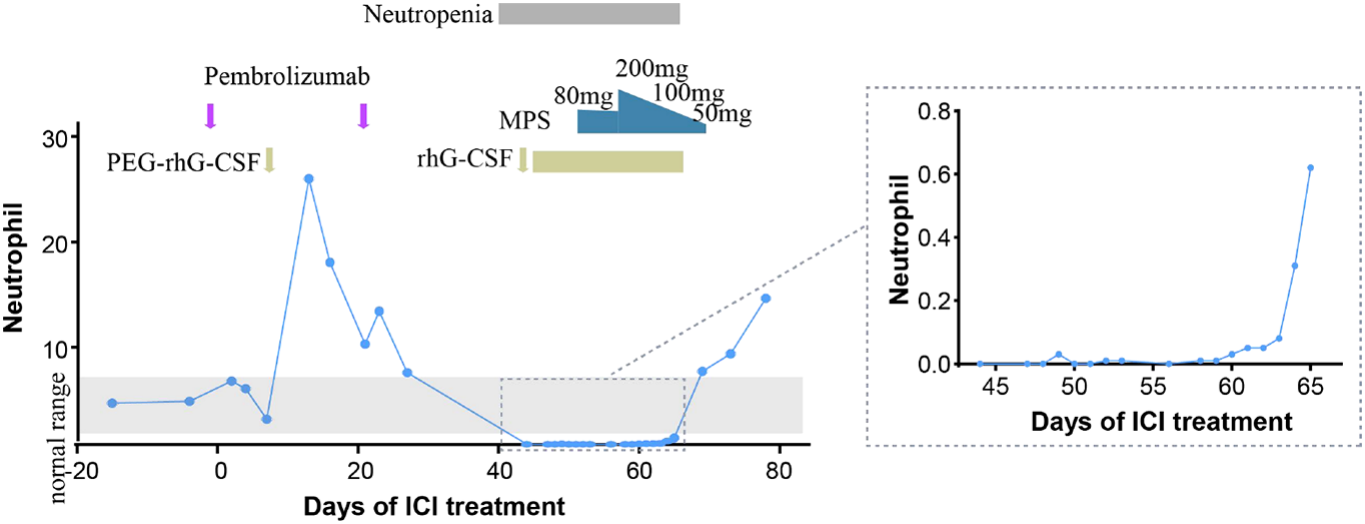
Timeline for absolute neutrophil count (ANC) with pembrolizumab administration. PEG-rhG-CSF, pegylated recombinant human granulocyte colony-stimulating factor; MPS, methylprednisolone.

**Figure 3:**
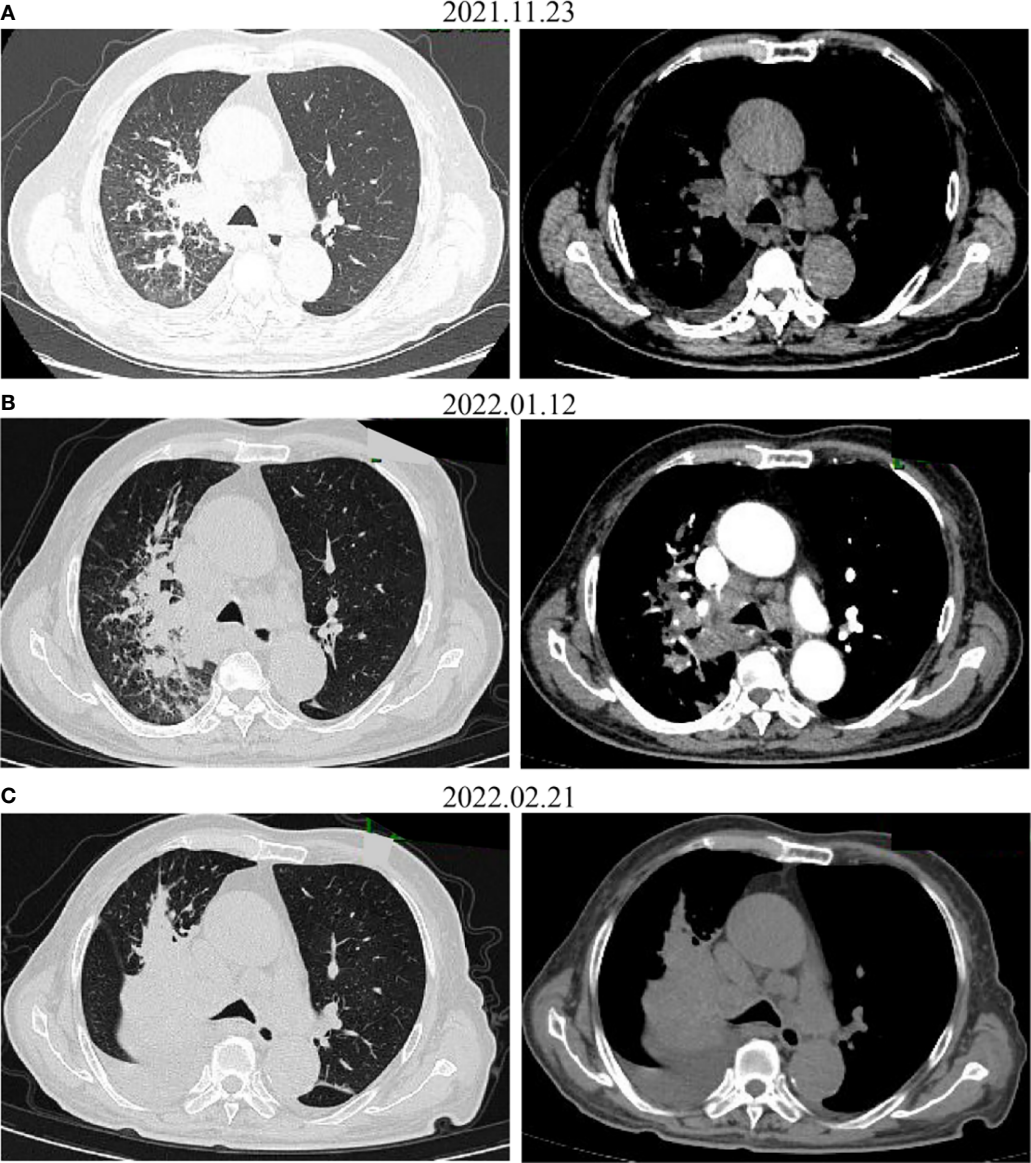
CT images before **(A)** and after administration of the first **(B)** and the second **(C)** dose with pembrolizumab. The tumor increased on CT.

The patient received one cycle of pembrolizumab (200 mg, day 1) and albumin-bound paclitaxel (nab-paclitaxel, 100 mg/m^2^, days 1 and 8), followed by brain tumor cyberknife radiation (30 Gy/2F). He was hospitalized in the cardiology department after the first cycle of treatment due to breathlessness and lower limb edema and was diagnosed with cardiac insufficiency (grade 2). Electrocardiogram (ECG) and echocardiography were applied; ECG showed sinus rhythm with STT changes similar to before chemoimmunotherapy, and echocardiography suggested enlarged left atrium, left ventricular systolic dysfunction, and no decrease in left ventricular ejection fraction (LVEF 52%). Serum evaluation of the patient during this period showed that the main abnormal factor was NT-ProBNP (3,840 pg/ml and eight times higher) but TNI was just slightly elevated (63.6 ng/L), CK-MB was 2 ng/ml (within the normal range), and LDH was 383 U/L (slightly elevated). The factors decreased quickly and the symptoms were relieved after medical treatment. Considering the intolerability of chemoimmunotherapy of the patient, the potential cardiotoxicity of nab-paclitaxel, and the patient’s situation, intravenous chemotherapy was stopped and changed to anlotinib hydrochloride capsules (12 mg days 1 to 14) in combination with pembrolizumab (200 mg). Forty-four days after the first administration and two therapy cycles of pembrolizumab, grade 4 neutropenia categorized by Common Terminology Criteria for Adverse Events (CTCAE 5.0) was detected (**Figure 2**). A complete blood count showed as follows: white blood cell: 500/μl, neutrophils: 0/μl, Hb: 10.7g/dl, and platelets: 19.7 × 10^4^/μl (**Table 1**). The tumor progressed after pembrolizumab treatment per imaging evaluation (**Figure 3**). He was hospitalized and treated with continuous recombinant human granulocyte stimulating factor injection (rhG-CSF, 300 mg, bid) for 11 days; however, neutropenia continued to deteriorate, and the timeline for absolute neutrophil count (ANC) with pembrolizumab administration is shown in **Table 1**. After 11 days of continuous rhG-CSF treatment without any improvement, a bone marrow aspiration was performed.

**Table 1:** The timeline of blood count and IL6.

Date	Days of ICI treatment	WBC (10^9^/L)	ANC (10^9^/L)	Hb (g/L)	PLT (10^9^/L)	Monocyte 10^9^/L)	Lymphocyte (10^9^/L)	IL6 (pg/ml)	ALT (U/L)	AST (U/L)	Cr (μmol/L)	BUN (mmol/L)	CRP (mg/L)	PCT (ng/L)
**2021.12.7**	−15	5.59	3.9	118	259	0.53	0.76		7	18	95.4	5.24		
**2021.12.18**	-4	6.23	4.08	125	223	0.52	1.21							
**2021.12.22**	0 (1^st^)													
**2021.12.24**	2	7.24	6	112	213	0.2	0.9							
**2021.12.26**	4	6.27	5.28	110	218	0.16	0.63							
**2021.12.29**	7	3.4	2.39	112	270	0.18	0.78						11.5	
**2022.1.4**	13	28.18	25.22	116	251	1.3	1.21						55.68	
**2022.1.7**	16	21.03	17.27	108	241	1.61	1.9		14	19	88.5		29.04	
**2022.1.12**	21	11.15	9.51	107	269	0.58	0.95							
**2022.1.13**	22(2^nd^)													
**2022.1.14**	23	14.62	12.63	111	341	0.86	1.06							3.97
**2022.1.18**	27	8.59	6.82	102	366	0.72	0.95				142.4	16.05		
**2022.2.4**	**44**	**0.5**	**0**	107	197	0.02	0.5		17	22	95.5	7.84		
**2022.2.7**	47	0.31	0	100	173	0	0.3		18	23	123.4	6.76	45.19	
**2022.2.8**	48	0.36	0	102	193	0.01	0.3							
**2022.2.9**	49	0.46	0.03	106	201	0.01	0.42				126.9	6.56		
**2022.2.10**	50	0.49	0	106	206	0.01	0.47							
**2022.2.11**	51	0.51	0	103	223	0.01	0.49							
**2022.2.12**	52	0.47	0.01	109	264	0.01	0.45		12	16	92.8	6.46		
**2022.2.13**	53	0.46	0.01	104	279	0	0.45	129.69						
**2022.2.16**	56	0.44	0	100	289	0.06	0.4		17	24	103.2	7.06	78.7	0.29
**2022.2.18**	58	0.46	0.01	99	310	0.05	0.4							
**2022.2.19**	59	0.43	0.01	96	319	0.04	0.37							0.35
**2022.2.20**	60	0.44	0.03	94	351	0.09	0.32							
**2022.2.21**	61	0.53	0.05	98	448	0.09	0.39	76.53						
**2022.2.22**	62	0.59	0.05	96	470	0.14	0.4							
**2022.2.23**	63	0.62	0.08	97	457	0.18	0.36							
**2022.2.24**	64	0.93	0.31	109	519	0.33	0.29							
**2022.2.25**	65	1.17	0.62	109	413	0.31	0.24	6.61						0.38
**2022.3.1**	**69**	**7.68**	**6.94**	107	130	0.4	0.33							
**2022.3.5**	73	9.42	8.59	96	56	0.39	0.43				133.5		6.92	
**2022.3.10**	78	14.53	13.87	74	371	0.34	0.31				82.5		85.7	

Hb, hemoglobin; PLT, platelets; WBC, white blood cells; ANC, absolute neutrophil count; ALT, alanine aminotransferase; AST, aspartate transaminase; Cr, creatinine; BUN, blood urea nitrate; CRP, C-reactive protein; PCT, procalcitonin. Bold values provided in table shows the date of neutropenia occurred and recovered.

Fifty-eight days after the first administration of pembrolizumab and after 11 days of rhG-CSF treatment, a complete blood count showed the following: white blood cell: 460/μl, neutrophils: 0/μl, Hb: 9.9 g/dl, and platelets: 310 × 10^3^/μl (**Table 1**). He also had hypoproteinemia (ALB 24.9 g/ml), liver function was normal [aspartate aminotransferase (AST): 36 U/L, alanine aminotransferase (ALT): 28 U/L, and alkaline phosphatase (ALP): 64 U/L, lactate dehydrogenase (LDH,127 U/L) and procalcitonin (PCT, 0.35) levels were normal. Renal function was slightly abnormal with a creatinine of 149.9 μmol/L and a glomerular filtration rate of 37.9 ml/min. The coagulation system parameters were normal, but after continuous neutropenia status, the sputum culture was positive for *Staphylococcus haemolyticus*, and the fungal GM test was positive. No evidence suggested infection of HAV, HBV, HCV, HEV, HSV, CMV, EBV, or HPV. The vital signs of the patient were normal with no fever. Physical examination showed lower limb edema. The patient continued using anti-hypertension, anti-diabetes, and anti-hyperlipidemia drugs (levamlodipine, sacubitril valsartan sodium tablets, dapagliflozin, aspirin, clopidogrel, and atorvastatin) alongside the anti-cancer treatment.

**Figure 4:**
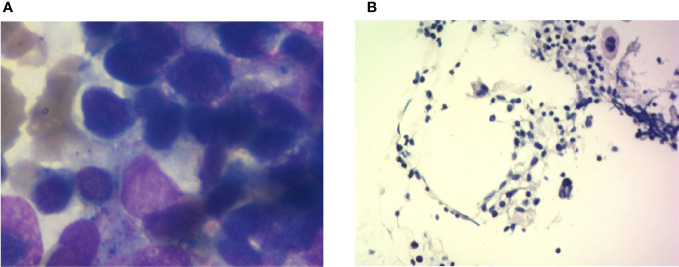
Bone marrow imaging. **(A)** Bone marrow cytological smear; there is no malignant tumor invasion into the bone marrow. **(B)** Bone marrow biopsy; the specimen shows agranulocytosis.

The bone marrow smear showed that neutrophils are rare, and the biopsy showed that megakaryocytes can be easily seen and that there was no evidence of myelodysplasia. Malignant tumor infiltration to bone marrow was not present (**Figure 4**, **Table 2**). Autoimmune disease detection showed that anti-nuclear antibody (ANA) was positive with a titer of 1:100; other items were normal. After 14 days of G-CSF treatment, the neutrophil of the patient did not have improvement, and myeloid metastasis was excluded through a bone marrow smear; the patient was taken into consideration for ICI-related neutropenia.

**Table 2:** The results of bone marrow cytology smear.

Cells		Blood smear	Bone marrow smear		
			Average	± SD	%
**Primitive blood cells**			0.08	0.01	
**Granulocyte lineage**	Primitive granulocytes		0.64	0.33	0.5
	Promyelocyte		1.57	0.60	
	Neutrophilic		6.49	2.04	
	Myelocyte		7.90	1.97	
	Metamyelocyte		23.72	3.50	
	Stab granulocyte		9.44	2.92	
	Segmented granulocyte				
	Eosinophilic		0.38	0.23	
	Myelocyte		0.49	0.32	
	Metamyelocyte		1.25	0.61	
	Stab granulocyte		0.86	0.61	
	Segmented granulocyte				
	Basophilic		0.02	0.05	
	Myelocyte		0.06	0.07	
	Metamyelocyte		0.10	0.09	
	Stab granulocyte		0.03	0.05	0.50
	Segmented granulocyte				
**Erythroid lineage**	Normoblast		0.57	0.30	1
	Basophilic normoblast		0.92	0.41	3
	Polychromatic normoblast		7.41	1.91	20.50
	Orthochromatic normoblast		10.75	2.36	41.50
**Lymphocytes**	Lymphoblast		0.05	0.09	
	Prolymphocyte		0.47	0.84	
	Mature lymphocyte	100	22.78	7.04	30.00
**Monocyte**	Monocyte		0.01	0.04	
	Promonocyte		0.14	0.19	
	Mature monocyte		3	0.88	
**Plasma cell**	Plasmablast		0.004	0.22	
	Proplasmacyte		0.104	0.16	
	Mature plasmacyte		0.71	0.42	1.00
**Others**	Reticulocytes		0.05	0.09	2.00
	Unknown		0.03	0.09	
**Granulocyte lineage/Erythroid lineage 3-5/1**					

On the 59th day post-first administration of pembrolizumab, he was treated with intravenous methylprednisolone sodium succinate (MPS; 80 mg/day for 5 days) firstly, which showed a very slow effect. Then, the dose was changed to 200 mg/day for 3 days and 100 mg/day for 3 days, followed by oral prednisolone (PSL; 50 mg/day, cut into half every 3 days). At the same time, rhG-CSF, antibiotic, and antifungal drugs were treated. After 69 days of the first administration of pembrolizumab, the neutrophil count returned to normal (white blood cell: 7,680/μl, neutrophils: 6,940/μl, Hb:10.7 g/dl, and platelets: 13×10^4^/μl). However, he developed severe cerebral infarction, which progressed rapidly, and he was, therefore, referred to the neurology department and died because of neurological problems.

## Discussion

The hematological irAE is a rare immune side effect; in recent times, coupled with over-expanding approvals for new ICI products and indications, there has been a gradual increase in related reports. Several guidelines recommend in detail the management of immunotherapy-related toxicities, including the American Society for Clinical Oncology (ASCO) (6), the European Society for Medical Oncology (ESMO) (7), the National Comprehensive Cancer Network (NCCN) (8), the consensus statement from the Society for Immunotherapy of Cancer (SITC) (9), and the Chinese Society of Clinical Oncology (CSCO). However, there are currently no uniform diagnostic and management criteria for immune-related hematologic side effects; hence, conducting relevant reports and case studies will help us to understand and standardize clinical management.

In ICI-related hematological toxicity, anemia and thrombocytopenia are the most common adverse events; neutropenia is rare among hematological irAEs. The median time of occurrence for neutropenia was 10.5 weeks after the first administration of ICI treatment (2.2–25.4 weeks) (10); PD-1/PD-L1 inhibitors are more frequent than CTLA4 inhibitors. Here, we report a case of neutropenia after 6.3 weeks (44 days) of the first administration of pembrolizumab and recovery after 9.9 weeks (69 days). The diagnostic principles and mechanisms for ICI-related neutropenia are still not clear. We should exclude other drug interference, check autoimmune indexes, and perform bone marrow aspiration (11). In this present case, nab-paclitaxel was conducted with ICI treatment in the first cycle; myelosuppressive toxicity caused by albumin paclitaxel often occurred in 1–2 weeks and recovered in 3 weeks, which was not fit for this patient. Bone marrow aspiration did not show myelodysplastic disease, and immunological parameters before the first cycle therapy of the patient showed an ANA positive test. A study showed that the presence of preexisting antibodies was associated with the development of irAEs in NSCLC with nivolumab or pembrolizumab (12); it is possible that some specific autoantibodies targeting neutrophils in serum are activated, leading to autoimmune neutropenia, but the more detailed mechanisms involved and dynamic autoantibody changes need to be further explored. Moreover, a study reported that 9% of the patients with hem-irAEs had a concomitant history of lymphocytic leukemia (4), suggesting that patients with mature lymphoid B clones may be at an increased risk for hematologic immunotoxicity; a lymphocytic infiltrate detection could provide evidence. More detailed mechanisms of ICI-related neutropenia need to be further explored. In this case, anlotinib and radiotherapy were conducted following the first administration of pembrolizumab, and a previous study showed that 4.3% grade 3/4 neutropenia occurred in recurrent or advanced endometrial cancer patients treated with sintilimab plus anlotinib (13); whether anti-angiogenic agents and radiotherapy increased the probability of neutropenia remains to be determined. However, considering the long duration of the patient’s neutropenia and lack of response to G-CSF, only one cycle of anlotinib and small radiation volume of brain tumor cyberknife radiation, immune factors were considered to be the main cause.

The management of neutropenia has not been detailed and clarified by ESMO or NCCN/ASCO/CSCO guidelines; SITC recommends treatment including steroids (prednisone 1 mg/kg oral or equivalent) together with G-CSF (9), but the time and duration of steroids were not clear. J.M. Michot reviewed the hematological irAEs and suggested systematical corticosteroid application without any firm evidence of efficacy, as they could accentuate the risk of infection (11). Infection is one of the most severe complications along with neutropenia, Boegeholz did one of the largest literature reviews of immune checkpoint inhibitor-related neutropenia; 3 patients (13%) had a bacterial infection, but none had a fungal infection (10). In this case, the patient got a positive result from the sputum culture and GM test; thus, antibacterial and antifungal drugs were administered along with glucocorticoids. This case adds to the growing body of evidence for bacterial and fungal infection following immune-related neutropenia after ICI therapy. Since immune-related neutropenia and infection are life-threatening, sputum culture and blood culture are recommended after discontinuation of ICIs. The application of antibiotics can affect the distribution of gut microbiota, leading to resistance to anti-PD-1 inhibitors (14). One study suggested that antibiotic therapy before but not concurrently with ICI is associated with worse treatment response and OS in cancer patients (15). How to balance the relationship between the anti-infective application for side effects treatment and clinical anti-tumor efficacy is a problem worthy of our consideration.

Since a neutropenia-complicated infection may be fatal, more attention needs to be paid to predictive biomarkers of adverse reactions to ICI treatment. IL6 is a cytokine that was reported to increase anti-PD-1-related dermatological toxicity (16, 17); severe AE rate in NSCLC patients receiving anti-PD-1 treatment was higher in the IL6 elevated group (18), while decreased IL6 level was reported to be associated with remission of colitis (19). In our case, the IL6 level was decreased in the recovery process of neutropenia, with 129.69 pg/ml on the 53rd day, 76.53 pg/ml on the 61st day, and 6.61 pg/ml on the 65th day after the first administration of pembrolizumab (**Table 2**). On the 69th day, the neutrophil count returned to normal. Our case gives a clue that a decreased level of IL6 may be an efficient biomarker for the remission of neutropenia, but there was no baseline IL6 level available for comparison. Additionally, IL6 can be released with growth factors into the bone marrow microenvironment (20); evaluation of IL6 level in the bone marrow might be another effective way. The predictive role of IL6 needs further validation in large-cohort studies and the cutoff of high level needs further definition.

Several studies have indicated that irAEs were associated with the efficacy of anti-PD-1 treatment in multiple tumor types, like NSCLC (21), melanoma (22), and gastric cancer (23). In hepatocellular carcinoma (HCC), the results show that the non-irAEs group was associated with independently poor prognosis, and low-grade irAE was predictable for better treatment efficiency (24). A pooled analysis of 1,747 patients with advanced urothelial cancer showed that an immune-mediated adverse event (imAE) occurred in 28% who did respond to the study drug, but 12% did not respond (25). A prospective cohort study from Japan showed that early irAEs were associated with a better outcome after nivolumab in NSCLC (26). A prospective cohort study with 73 NSCLC patients who received anti-PD-1 therapy (nivolumab or pembrolizumab) showed that 25 (34.2%) developed autoimmune skin toxic effects, which were more frequent in patients with complete remission or partial remission (68.2%) than those with progressive or stable disease (19.6%) (21). In this case, TPS for PD-L1 staining of the patient was 40% positive and he developed irAE, but after two cycles of anti-PD-1 treatment, a CT scan showed disease progression (**Figure 3**), which indicated the need for further research on the mechanism of ICI-mediated autoimmune toxic effects and the correlation with response to therapy. Various factors can affect the effectiveness of immunotherapy including age, immunity, and tumor-infiltrating lymphocytes (TILs), indicating that multi-element predictive models rather than single markers would better predict outcomes. Such an understanding will help us to better control adverse effects and further improve immunotherapy.

In conclusion, our case demonstrates that grade 4 neutropenia associated with ICIs, although rare, is often severe and presents with infectious complications. Biomarkers including IL6 to early identify ICI-associated neutropenia and timely intervention with immunosuppression and G-CSF may alleviate the duration and thus prevent a potentially fatal outcome. Though there were still some defects and deficiencies during the process, such as bone penetration, glucocorticoid therapy should be carried out earlier, and the combination of intravenous immunoglobin (IVIG) therapy might benefit the patient. Our case adds to the growing body of evidence on the hematological immune adverse effect profiles of ICIs, and we would like to provide additional evidence and experience for the later diagnosis and treatment work in this immunotherapy era.

## Data availability statement

The original contributions presented in the study are included in the article/supplementary material. Further inquiries can be directed to the corresponding authors.

## Author contributions

LJC and XRD designed the study. QYT, LCL, YH and LJC collected the data. QYT and LCL analyzed and interpreted the data. QYT and LCL wrote the manuscript. LJC and XRD revised the manuscipt. All authors reviewed the manuscript and approved the final version for publication.

## Funding

This study was supported by the New National Natural Science Foundation of China (82200212) and the Bethune-Cancer Radiotherapy Translational Medicine Research Fund of China (Grant No. flzh202117).

## Conflict of interest

The authors declare that the research was conducted in the absence of any commercial or financial relationships that could be construed as a potential conflict of interest.

## Publisher’s note

All claims expressed in this article are solely those of the authors and do not necessarily represent those of their affiliated organizations, or those of the publisher, the editors and the reviewers. Any product that may be evaluated in this article, or claim that may be made by its manufacturer, is not guaranteed or endorsed by the publisher.

## References

[1] Yap TA Parkes EE Peng W Moyers JT Curran MA Tawbi HA . Development of immunotherapy combination strategies in cancer. Cancer Discovery (2021) 11:1368–97. doi: 10.1158/2159-8290.CD-20-1209 PMC8178168 33811048

[2] Dolladille C Ederhy S Sassier M Cautela J Thuny F Cohen AA . Immune checkpoint inhibitor rechallenge after immune-related adverse events in patients with cancer. JAMA Oncol (2020) 6:865–71. doi: 10.1001/jamaoncol.2020.0726 PMC7163782 32297899

[3] Martins F Sofiya L Sykiotis GP Lamine F Maillard M Fraga M . Adverse effects of immune-checkpoint inhibitors: Epidemiology, management and surveillance. Nat Rev Clin Oncol (2019) 16:563–80. doi: 10.1038/s41571-019-0218-0 31092901

[4] Delanoy N Michot JM Comont T Kramkimel N Lazarovici J Dupont R . Haematological immune-related adverse events induced by anti-PD-1 or anti-PD-L1 immunotherapy: A descriptive observational study. Lancet Haematol (2019) 6:e48–57. doi: 10.1016/S2352-3026(18)30175-3 30528137

[5] Liu C Ding L Zhu YH Chen C . A rare case of lung carcinoma acquires multidrug-resistant klebsiella pneumoniae pneumonia radiologically mimicking metastasis caused by nivolumab therapy-associated neutropenia. Ther Clin Risk Manage (2017) 13:1375–7. doi: 10.2147/TCRM.S144681 PMC5644596 29066905

[6] Schneider BJ Naidoo J Santomasso BD Lacchetti C Adkins S Anadkat M . Management of immune-related adverse events in patients treated with immune checkpoint inhibitor therapy: ASCO guideline update. J Clin Oncol (2021) 39:4073–126. doi: 10.1200/JCO.21.01440 34724392

[7] Haanen J Carbonnel F Robert C Kerr KM Peters S Larkin J . Management of toxicities from immunotherapy: ESMO clinical practice guidelines for diagnosis, treatment and follow-up. Ann Oncol (2018) 29:iv264–iv6. doi: 10.1093/annonc/mdy162 29917046

[8] Thompson JA Schneider BJ Brahmer J Achufusi A Armand P Berkenstock MK . Management of immunotherapy-related toxicities, version 1.2022, NCCN clinical practice guidelines in oncology. J Natl Compr Canc Netw (2022) 20:387–405. doi: 10.6004/jnccn.2022.0020 35390769

[9] Brahmer JR Abu-Sbeih H Ascierto PA Brufsky J Cappelli LC Cortazar FB . Society for immunotherapy of cancer (SITC) clinical practice guideline on immune checkpoint inhibitor-related adverse events. J Immunother Cancer (2021) 9:e002435. doi: 10.1136/jitc-2021-002435 34172516 PMC8237720

[10] Boegeholz J Brueggen CS Pauli C Dimitriou F Haralambieva E Dummer R . Challenges in diagnosis and management of neutropenia upon exposure to immune-checkpoint inhibitors: Meta-analysis of a rare immune-related adverse side effect. BMC Cancer (2020) 20:300. doi: 10.1186/s12885-020-06763-y 32290812 PMC7155336

[11] Michot JM Lazarovici J Tieu A Champiat S Voisin AL Ebbo M . Haematological immune-related adverse events with immune checkpoint inhibitors, how to manage? Eur J Cancer (2019) 122:72–90. doi: 10.1016/j.ejca.2019.07.014 31634647

[12] Toi Y Sugawara S Sugisaka J Ono H Kawashima Y Aiba T . Profiling preexisting antibodies in patients treated with anti-PD-1 therapy for advanced non-small cell lung cancer. JAMA Oncol (2019) 5:376–83. doi: 10.1001/jamaoncol.2018.5860 PMC6439838 30589930

[13] Wei W Ban X Yang F Li J Cheng X Zhang R . Phase II trial of efficacy, safety and biomarker analysis of sintilimab plus anlotinib for patients with recurrent or advanced endometrial cancer. J Immunother Cancer (2022) 10:e004338. doi: 10.1136/jitc-2021-004338 35623659 PMC9150151

[14] Derosa L Hellmann MD Spaziano M Halpenny D Fidelle M Rizvi H . Negative association of antibiotics on clinical activity of immune checkpoint inhibitors in patients with advanced renal cell and non-small-cell lung cancer. Ann Oncol (2018) 29:1437–44. doi: 10.1093/annonc/mdy103 PMC6354674 29617710

[15] Pinato DJ Howlett S Ottaviani D Urus H Patel A Mineo T . Association of prior antibiotic treatment with survival and response to immune checkpoint inhibitor therapy in patients with cancer. JAMA Oncol (2019) 5:1774–8. doi: 10.1001/jamaoncol.2019.2785 PMC6743060 31513236

[16] Tanaka R Okiyama N Okune M Ishitsuka Y Watanabe R Furuta J . Serum level of interleukin-6 is increased in nivolumab-associated psoriasiform dermatitis and tumor necrosis factor-alpha is a biomarker of nivolumab recativity. J Dermatol Sci (2017) 86:71–3. doi: 10.1016/j.jdermsci.2016.12.019 28069323

[17] Valpione S Pasquali S Campana LG Piccin L Mocellin S Pigozzo J . Sex and interleukin-6 are prognostic factors for autoimmune toxicity following treatment with anti-CTLA4 blockade. J Transl Med (2018) 16:94. doi: 10.1186/s12967-018-1467-x 29642948 PMC5896157

[18] Ozawa Y Amano Y Kanata K Hasegwa H Matsui T Kakutani T . Impact of early inflammatory cytokine elevation after commencement of PD-1 inhibitors to predict efficacy in patients with non-small cell lung cancer. Med Oncol (2019) 36:33. doi: 10.1007/s12032-019-1255-3 30825015

[19] Yoshino K Nakayama T Ito A Sato E Kitano S . Severe colitis after PD-1 blockade with nivolumab in advanced melanoma patients: Potential role of Th1-dominant immune response in immune-related adverse events: two case reports. BMC Cancer (2019) 19:1019. doi: 10.1186/s12885-019-6138-7 31664934 PMC6819390

[20] Harmer D Falank C Reagan MR . Interleukin-6 interweaves the bone marrow microenvironment, bone loss, and multiple myeloma. Front Endocrinol (Lausanne) (2018) 9:788. doi: 10.3389/fendo.2018.00788 30671025 PMC6333051

[21] Berner F Bomze D Diem S Ali OH Fassler M Ring S . Association of checkpoint inhibitor-induced toxic effects with shared cancer and tissue antigens in non-small cell lung cancer. JAMA Oncol (2019) 5:1043–7. doi: 10.1001/jamaoncol.2019.0402 PMC6487908 31021392

[22] Nakamura Y Tanaka R Asami Y Teramoto Y Imamura T Sato S . Correlation between vitiligo occurrence and clinical benefit in advanced melanoma patients treated with nivolumab: A multi-institutional retrospective study. J Dermatol (2017) 44:117–22. doi: 10.1111/1346-8138.13520 27510892

[23] Matsuoka H Hayashi T Takigami K Imaizumi K Shiroki R Ohmiya N . Correlation between immune-related adverse events and prognosis in patients with various cancers treated with anti PD-1 antibody. BMC Cancer (2020) 20:656. doi: 10.1186/s12885-020-07142-3 32664888 PMC7362440

[24] Xu S Lai R Zhao Q Zhao P Zhao R Guo Z . Correlation between immune-related adverse events and prognosis in hepatocellular carcinoma patients treated with immune checkpoint inhibitors. Front Immunol (2021) 12:794099. doi: 10.3389/fimmu.2021.794099 34950153 PMC8691363

[25] Maher VE Fernandes LL Weinstock C Tang S Agarwal S Brave M . Analysis of the association between adverse events and outcome in patients receiving a programmed death protein 1 or programmed death ligand 1 antibody. J Clin Oncol (2019) 37:2730–7. doi: 10.1200/JCO.19.00318 31116675

[26] Teraoka S Fujimoto D Morimoto T Kawachi H Ito M Sato Y . Early immune-related adverse events and association with outcome in advanced non-small cell lung cancer patients treated with nivolumab: A prospective cohort study. J Thorac Oncol (2017) 12:1798–805. doi: 10.1016/j.jtho.2017.08.022 28939128

